# Increased Absorption and Inhibitory Activity against *Candida* spp. of Imidazole Derivatives in Synergistic Association with a Surface Active Agent

**DOI:** 10.3390/microorganisms12010051

**Published:** 2023-12-27

**Authors:** Florin Aonofriesei

**Affiliations:** Department of Natural Sciences, Faculty of Natural and Agricultural Sciences, “Ovidius” University of Constanța, 1 University Street, 900470 Constanța, Romania; aonofriesei_florin@yahoo.fr

**Keywords:** imidazole derivatives, *Candida* spp., SDS, synergistic association

## Abstract

This paper’s purpose was to evaluate the interaction between three imidazole derivatives, (2-methyl-1*H*-imidazol-1-yl)methanol (SAM3), 1,1′-methanediylbis(1*H*-benzimidazole (AM5) and (1*H*-benzo[d]imidazol-1-yl)methanol 1-hydroxymethylbenzimidazole (SAM5) on the one hand, and sodium dodecyl sulphate (SDS) on the other, as antifungal combinations against *Candida* spp. Inhibitory activity was assessed using the agar diffusion method and Minimal Inhibitory Concentration (MIC) and showed moderate inhibitory activity of single imidazole derivatives against *Candida* spp. The mean value of MIC ranged from 200 µg/mL (SAM3) to 312.5 µg/mL (SAM3), while for SDS the MIC was around 1000 µg/mL. When used in combination with SDS, the imidazole derivatives demonstrated an improvement in their antifungal activity. Their MIC decreased over five times for AM5 and over seven times for SAM3 and SAM5, respectively, and ranged from 26.56 µg/mL (SAM3) to 53.90 µg/mL (AM5). Most combinations displayed an additive effect while a clear synergistic effect was recorded in only a few cases. Thus, the FIC Index (FICI) with values between 0.311 and 0.375 showed a synergistic effect against *Candida* spp. when SDS was associated with SAM3 (three strains), SAM5 (two strains) and AM5 (one strain). The association of imidazole derivatives with SDS led to the increased release of cellular material as well as the intracellular influx of crystal violet (CV), which indicated an alteration of the membrane permeability of *Candida* spp. cells. This favored the synergistic effect via increasing the intracellular influx of imidazoles.

## 1. Introduction

Fungal infections have become more frequent worldwide every year, accounting for millions of patients and a high death toll [1,2]. *Candida albicans* and non-albicans strains are by far the most significant pathogens among fungal infections [3,4]. The mortality rate due to *Candida* spp. infections is high, especially in elderly and immunocompromised patients. The treatments of *Candida* spp. infections are often associated with significant costs [5]. *Candida* spp. is an opportunistic pathogen that can exist also as a commensal in healthy individuals without any apparent symptoms. It can become a potent pathogen under particular conditions, causing superficial and invasive infections due to diverse virulence traits such as biofilm formation, specific hydrolases and morphological changes [4]. In recent years, many antifungal drugs have become ineffective as more and more resistant strains have emerged [6,7]. This is a major medical concern because there are only a few treatment options, limited to only several classes of antifungals [8]. As a result, there is an urgent need to find new active compounds, or at least combinations of old compounds, efficient for controlling *Candida* spp. infections. Data on the synthesis of SAM3, AM5 and SAM5 have been previously published [9,10], including some aspects related to their antibacterial properties [11,12]. Moreover, in two more recent articles, we tried to associate some of these compounds with other active substances in order to find more effective combinations against pathogenic microorganisms [13,14]. Most of the results were encouraging to further explore new combinations with significant antimicrobial potential. Because imidazoles are well-known for their antifungal potential [15,16,17,18] we thought it would be useful to test some new imidazole derivatives against *Candida* strains isolated from clinical specimens. At the same time, we also found it interesting to associate these derivatives with SDS in order to know if we could improve their inhibitory potential. Surfactants are amphipathic molecules that can interact with microbial cell structures. One such molecule is SDS, which also displays a weak antifungal activity [19]. Its efficacy depends on the type of cells on which it acts, whether they are planktonic or embedded in a biofilm structure [20]. SDS is especially effective in preventing the formation of *Candida albicans* biofilms [21]. The association of SDS with other organic compounds has been shown to be effective as sanitizing agents in some foods [22]. There are also reports showing that SDS may increase the activity of some antifungal azoles, such as fluconazole [19]. Therefore, as a continuation of our work, we thought it would be useful to associate SAM3, AM5 and SAM5 with SDS and to test these combinations against *Candida* spp. The results showed that, in many cases, the activity of associations was unexpectedly better than that of compounds used alone, and this fact can be attributed to the increased membrane permeability caused by the presence of sublethal doses of SDS.

## 2. Materials and Methods

### 2.1. Experimental Part

Details regarding the protocol for the synthesis, structure and characterization of AM5, SAM3 and SAM5 have been previously published [9,10].

### 2.2. Candida Strains

The pathogenic strains have been isolated in several clinical laboratories (Constanta, Romania) from patients with different types of infections (Table 1). The reference strain was purchased from Microbiologics (St. Cloud, MN, USA). Stock solutions of imidazoles (10 mg/mL) were prepared in dimethyl sulphoxide (DMSO) 5% (*w*/*vol*), while SDS was dissolved in deionized water to reach 2% (*w*/*vol*) concentration.

### 2.3. Agar Diffusion Assay

Preliminary tests regarding the inhibitory activity of compounds were performed using the agar diffusion method. Several colonies from 24 h old cultures grown on Sabouraud Dextrose Agar (SDA) were suspended in sterile saline solution and adjusted to 1–5 × 10^5^ CFU/mL. Inoculations were made via flooding the SDA surface with 1 mL of fungal suspension. The excess of suspension was removed and plates were kept inverted to allow the agar to dry. Afterward, wells were punched on agar plates (5 mm in diameter), and 50 µL of each compound was pipetted into the wells. The plates were incubated at 37 °C for 48 h, and antifungal activity was assessed as mm of inhibition zones. In order to compare the susceptibility of *Candida* spp. to imidazole derivatives SAM3 (200 µg), AM5 (300 µg) and SAM5 (300 µg), we used fluconazole (25 µg, Oxoid—Basingstoke, UK), an antifungal widely used in the treatment of infections caused by *Candida* spp., as a positive control. The testing and evaluation of the strains’ susceptibility were performed according to the CLSI standards [23].

### 2.4. MIC Estimation

MIC was estimated using broth dilution according to a slightly modified EUCAST protocol [24]. Reference and clinical strains were sub-cultured on SDA and were grown in laboratory at 37 °C in Sabouraud Dextrose Broth (SDB). Inoculum was prepared by suspending 4–5 colonies (of about 1 mm in diameter) in sterile saline solution. Suspension was homogenized using a vortex shaker for 20 s. The density of inoculum was adjusted to 0.5 McFarland standard. Serial two-fold dilutions of the compounds were made in SDB to achieve a concentration between 39.06 µg/mL and 2500 µ/mL for SDS and between 7.81 µg/mL and 1000 µg/mL for imidazoles. MIC was estimated as the lowest concentration of compounds that completely inhibited the growth of fungal cells and expressed as µg/mL. All experiments were performed in triplicate using negative controls (non-inoculated broths) and positive controls (inoculated broths without imidazoles). Fluconazole powder (Pfizer, New York, NY, USA) was dissolved in sterile saline water (0.85% NaCl), and two-fold dilutions were made in SDB with a range of concentrations between 0.125 and 128 µg/mL. Inoculation and incubation were carried out under conditions similar to the experimental variants.

### 2.5. Checkboard Assay

In order to estimate the interaction between SDS and imidazoles, we used the slightly modified protocol following the guidelines described in the literature [25,26,27,28]. In a series of test tubes, two-fold dilutions of SDS and imidazole derivatives were prepared in SDB. The MICs obtained for each tested agent and bacteria were considered and dilutions ranged from 1/2 to 1/64 MIC. Concentration range for SDS varied between 2.5 mg/mL and 39.06 µg/mL, while for imidazoles it was from 500 µg/mL to 7.81 µg/mL. The concentration range of each agent was organized as follows: the concentration of SDS increased from left to right while imidazole derivative concentration decreased from top to bottom. The first two test tubes contained only one agent at MIC concentration, while another test tube served as a blank and received only the fungal suspension.

Fungal suspensions of 24 h old cultures were added to each range combination of the tested compound to reach a final density of 1–5 × 10^5^ CFU/mL. Inoculated test tubes were incubated at 37 °C for 48 h. *Candida* spp. growth was verified by visual inspection of turbidity. Each test was performed in triplicate. Interaction between SDS and imidazole derivatives was assessed as Fractional Inhibitory Concentration (FIC). FICs were calculated as follows: FIC_SDS_ = MIC of SDS in combination/MIC of SDS alone; FIC of SAM3 = MIC of SAM3 in combination/MIC of SAM3 alone. Similarly, FICs were calculated for other combinations of SDS + AM5 and SDS + SAM5. FIC Index (FICI) for a specific combination (e.g., SDS + SAM3) was calculated by adding FIC values of the two compounds: FICI = FIC_SDS_ + FIC_SAM3_. Interaction was assessed as follows: (i) synergism—FICI ≤ 0.5; (ii) additive or indifference—FICI = 0.5–4.0; (iii) antagonism—FICI > 4.

### 2.6. Fungicidal Activity over Time

To further investigate the effect of combinations between SDS and imidazoles, we used the time-kill procedure. Test tubes containing SDB and combinations of SDS + imidazoles were inoculated with overnight *Candida albicans* suspensions having a final density of 1–5 × 10^5^ CFU/mL. Inoculated tubes were incubated at 37 °C, and 100 µL of cultures were extracted at defined periods of time (0, 4, 8, 24 h) and inoculated on SDA. Each experimental variant was inoculated in triplicate. SDA plates were incubated for 48 h at 37 °C and colonies were counted. The mean value of colonies was then plotted against time.

### 2.7. Leakage of Nucleic Acids

To understand the contribution of SDS to membrane permeabilization and to the antifungal effect of the combination, we estimated the release of cellular material according to the previously published protocols [29,30]. Before the experiment, *Candida albicans* strains were grown in SDB for 48 h, collected via centrifugation (5000 rpm, 15 min) and washed three times with Phosphate Buffered Saline (PBS, pH = 7.4). They were later resuspended in PBS, their density was adjusted to 1 × 10^6^ CFU/mL and exposed to the action of SDS 2% and SAM3 (1, 2 and 4 MIC) for 2 h at 35 °C. As a negative control, a culture of *Candida* incubated in the same conditions without SDS or imidazoles was used. After incubation, the cultures were centrifuged (14,000 rpm, 10 min), the cells were removed, and the supernatants were collected and used to determine the absorbance at 260 nm. The absorbance of the cell-free supernatant from all experimental variants was read at 260 nm using a Jasco UV-vis spectrophotometer (Thermo Fisher Scientific, Oxford, UK). A separate reading was made for PBS, SDS and imidazoles at the same wavelength, and the values obtained were subtracted from the total value of the readings made for the experimental variants. For each variant (controls, compounds and combinations), the experiments were performed in triplicate, with the final results being the average value of the three readings. 

### 2.8. Crystal Violet Uptake Assay

Changes in membrane permeability of *Candida* spp. were estimated using CV uptake assays according to the protocols described by Biernasiuk et al. (2021) and Janeczko et al. (2022) [31,32], with slight modifications. *Candida* spp. cultures were grown overnight in SDB, collected via centrifugation (5000 rpm, 15 min) and subsequently washed in PBS. Their density was adjusted to 1 × 10^6^ CFU/mL in PBS and compounds SAM3 (1, 2 and 4 MIC), SAM5 (1, 2, and 4 MIC) and their combinations with SDS (1000 µg/mL) were added. Incubation was carried out at 35 °C for 5 h, and later the cultures were washed in PBS and centrifuged (5000 rpm, 15 min). The cultures were exposed to CV (10 µg/mL) dissolved in PBS for 10 min at 35 °C, then centrifuged (14,000 rpm, 10 min), and the supernatants were collected. The absorbance of the supernatants containing the remaining CV from all experimental variants was read at 590 nm using a UV-vis double beam spectrophotometer Jasco. The optical density of the freshly prepared CV solution at 590 nm was considered 100%. The amount of CV taken up by the cultures was determined using the formula [31]: % CV uptake = 100 − [(OD_590_ sample/OD_590_ CV solution) × 100].

### 2.9. Statistical Analysis 

Statistical analysis was carried out using paired-sample *t*-tests with STW Statistics 18 software (v. 9.08) to determine if the combinations had a significantly higher inhibitory effect vs. compounds alone. To estimate the inhibitory effect of the imidazoles from the experimental variants vs. controls, we used the Pearson product–moment correlation.

## 3. Results

The agar diffusion tests indicated a moderate activity of derivatives, a little higher in the case of SAM3 (Table 2), but lower than that recorded for fluconazole. The higher activity of SAM3 vs. AM5 and SAM5 suggested that the low hydrosolubility of benzimidazoles might influence their diffusion and effect on *Candida* spp. growth. Also, the lower activity of AM5 in comparison with SAM5 suggested that a larger hydrophobic molecule is less efficient in achieving optimal in vitro inhibitory concentrations. The results showed that the strains were resistant according to CLSI standards [23].

### 3.1. MIC Estimation

The antifungal activity of imidazole derivatives was lower compared to fluconazole, by 2.27 times (SAM3) and by more than 3 times (SAM5 and AM5) (Table 3). The tested derivatives showed a moderate inhibitory power with the mean value of MIC ranging between 200 µg/mL (SAM3) and 312.5 µg/mL (AM5). 

However, the individual strains manifested a significant variability of susceptibility to these derivatives. This was the case of SAM3, when MIC fluctuated between 62.5 µg/mL and 500 µg/mL in tested *Candida* strains.

SDS demonstrated the ability to inhibit the growth of *Candida* spp. at much higher concentrations of about 1 mg /mL (Table 3). SDS is an anionic detergent with inhibitory activity at relatively high concentrations. SDS interacts primarily with the plasma membrane and increases its permeability. It may penetrate through the pores of the cell wall and disrupt the membrane structure [33].

In some cases, there was an unexpected response of some strains to imidazoles and SDS. Thus, the MIC recorded for two strains (*Candida albicans* CAII3 and *Candida albicans* CAVI1) were high for imidazoles and unexpectedly low for SDS (Table 3). The variability of MIC could be related, probably, to the individual tolerance of the strains to imidazoles. All strains were recovered from infections (Table 1) and some of them might have been exposed to antifungals prior to isolation. Therefore, it was possible that some strains could acquire a certain degree of resistance against azole antifungals. As a result, they had a higher tolerance to the tested imidazoles. Benzimidazoles have a low solubility in water and this could be the reason for less efficacy when used in hydrophilic environments such as culture media. 

### 3.2. Antifungal Activity of Single and Associated Imidazoles with SDS

We considered synergism as having an FICI value below 0.5 and excluded the values very close to this threshold. The cases of synergism were relatively few and they were recorded only for four *Candida* strains (Table 4, Table 5 and Table 6). 

The other combinations manifested an additive effect, although some values were very close to that of the synergism threshold (Table 4, Table 5 and Table 6). The degree to which the synergistic effect was recorded seemed to be correlated ± with the mean value of MIC for the respective compounds (Table 3). The MIC differences between compounds used alone and in combination with SDS were statistically significant for the SAM3/SAM3 + SDS (*p* < 0.05) and SAM5/SAM5 + SDS (*p* < 0.05) pairs and less for the AM5/AM5 + SDS (*p* < 0.5) pair.

The association of imidazole derivatives with SDS has, in all cases, led to decreased MIC values and improved antimicrobial properties. When associated with SDS, the inhibitory power of derivatives increased 5.79 times for AM5, 7.49 times for SAM5 and 7.53 for SAM3. The effect of the association of imidazoles with SDS was variable both in terms of the type of compound and the behavior of *Candida* strains. Thus, in the case of SAM3/SDS, a synergistic effect was observed for *C. albicans* ATCC 10231, *C. albicans* CaVI2 and *C. albicans* CaVI4, while for the other two compounds, the synergistic effect was recorded only for two strains (SAM5) and one strain of *Candida* (AM5) (Table 3, Table 4 and Table 5).

### 3.3. Fungicidal Activity over Time of Single and Associated Compounds with SDS

**Figure 1:**
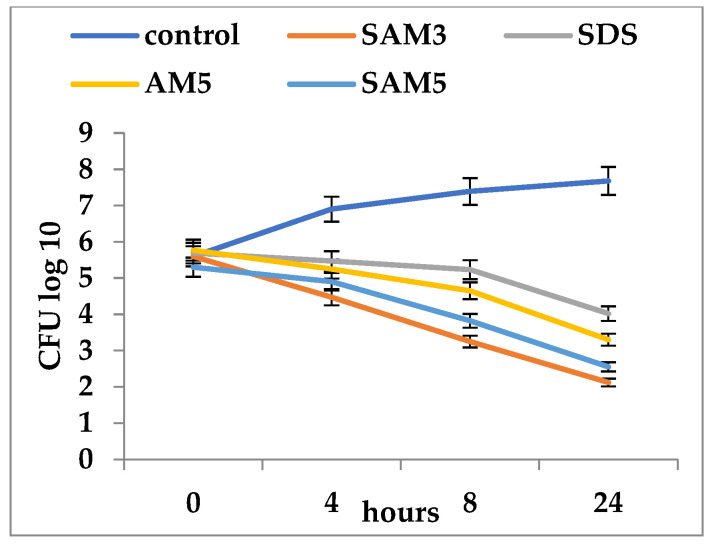
Time-kill of *Candida albicans* ATCC 10231 during exposure to imidazole derivatives and SDS (SAM3 = 500 µg/mL; AM5 = 500 µg/mL; SAM5 = 500 µg/mL; SDS = 2000 µg/mL).

When imidazoles were used unpaired with SDS, the most intense antifungal activity was recorded for SAM3, as a reduction in the viability of *C. albicans* ATCC cells from 5.6 log_10_ CFU/mL to 2.12 log_10_ CFU/mL (Figure 1). Antifungal activity was also shown with SAM5 (with a reduction in viability from 5.3. log_10_ CFU/mL to 2.55 log_10_ CFU/mL) and AM5 (reduction in viability from 5.77 log_10_ CFU/mL to 3.3 log_10_ CFU/mL) (Figure 1).

**Figure 2:**
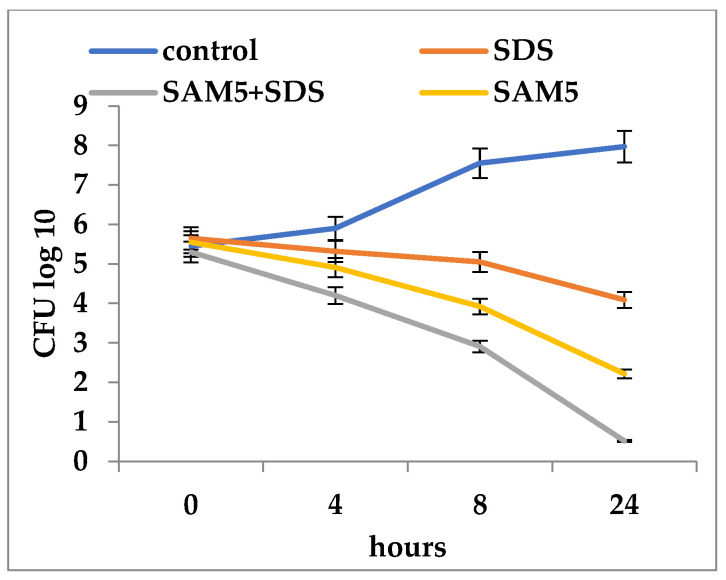
Time-kill of *Candida albicans* ATCC 10231 during exposure to imidazole derivatives and SDS (SAM5 = 500 µg/mL; SAM5 + SDS = 62.5 + 1250 µg/mL; SDS = 1500 µg/mL).

**Figure 3:**
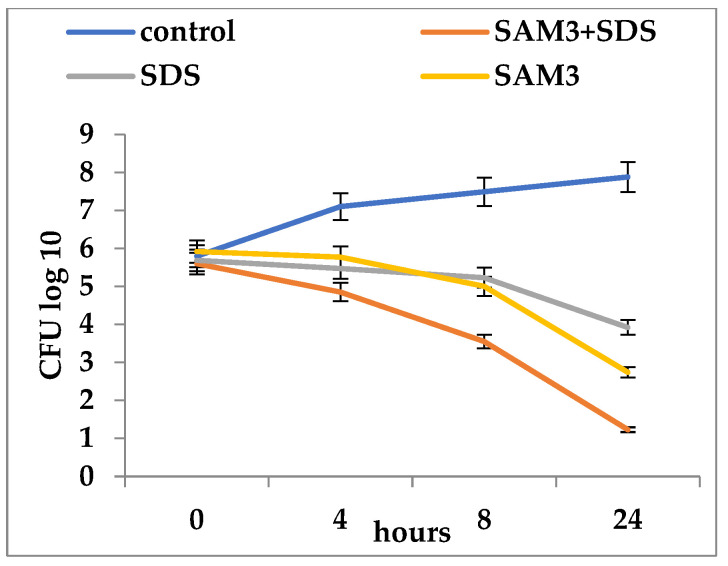
Time-kill of *Candida albicans* ATCC 10231 during exposure to imidazole derivatives and SDS (SAM3 = 500 µg/mL; SAM3 + SDS = 31.25 + 625 µg/mL; SDS = 1500 µg/mL).

**Figure 4:**
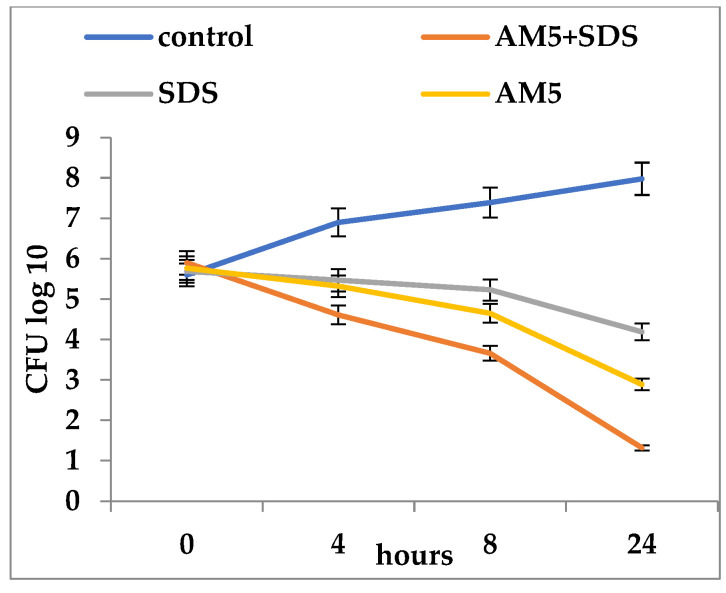
Time-kill of *Candida albicans* ATCC 10231 during exposure to imidazole derivatives and SDS (AM5 = 500 µg/mL; AM5 + SDS = 62.5 + 1250 µg/mL; SDS = 1500 µg/mL).

In general, the association of each of the three compounds with SDS increased the inhibitory activity at much lower concentrations than when they were used alone (Figure 2, Figure 3 and Figure 4). The association of SAM5 with SDS had an effect of increasing the mortality of *C. albicans* cells from 5.3 log_10_ CFU/mL to 0.52 log_10_ CFU/mL (Figure 2). When the SDS + SAM3 combination was used, an increased inhibitory effect was observed, although somewhat lower than in the previous case with a reduction in the number of viable *C. albicans* cells from 5.6 log_10_ CFU/mL to 1.23 log_10_ CFU/mL (Figure 3). The SDS + AM5 combination had a very similar inhibitory effect when a decrease in viable *C. albicans* cells was recorded from 5.9 log_10_ CFU/mL to 1.32 log_10_ CFU/mL (Figure 4). Comparatively, the most effective combination in terms of antifungal effect was SDS + SAM3 followed by the pairs SDS + SAM5 and SDS + AM5. It is worth noting that the inhibitory concentration of imidazoles drops a lot when they are used in combination with SDS.

When used alone, the most obvious antifungal effect was recorded for SAM3 (r = −0.89). Instead, in combination with SDS, the maximum inhibitory effect was observed for SAM5 + SDS (r = −0.93) and AM5 + SDS (r = −0.93) (Table 7).

### 3.4. Leakage of Nucleic Acids

**Figure 5:**
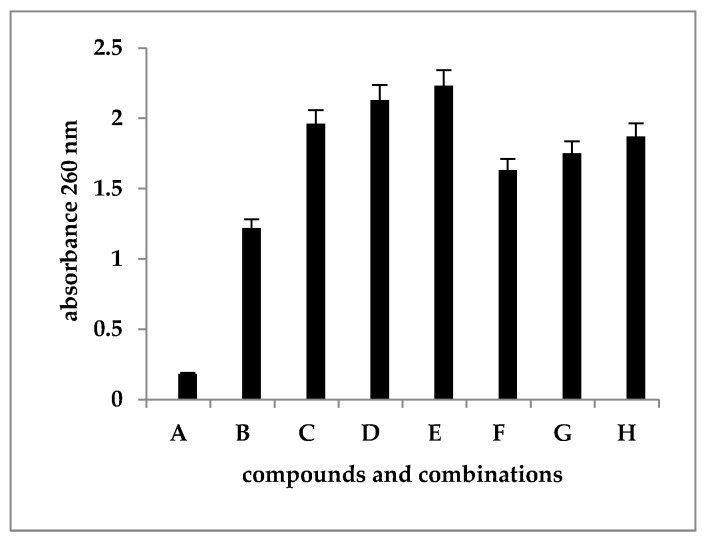
Leakage of cellular material in *C. albicans* cells detected as absorbance at 260 nm. A—negative control (cultures without compounds); B—positive control (SDS 2%); C—SAM3 1 MIC + SDS; D—SAM3 2 MIC + SDS; E—SAM3 4 MIC + SDS; F—SAM3 1 MIC; G—SAM3 2 MIC; H—SAM3 4 MIC.

The amount of cellular material released was obviously higher when imidazoles were associated with SDS, supporting the idea of synergistic action between the two groups of compounds (Figure 5). At the same time, the number of nucleic acids released from the cells increased with the increase in the concentration of imidazoles, which showed that these compounds were directly involved, along with SDS, in the disorganization and permeabilization of the cell membrane (Figure 5).

### 3.5. Crystal Violet Uptake Assay

**Figure 6:**
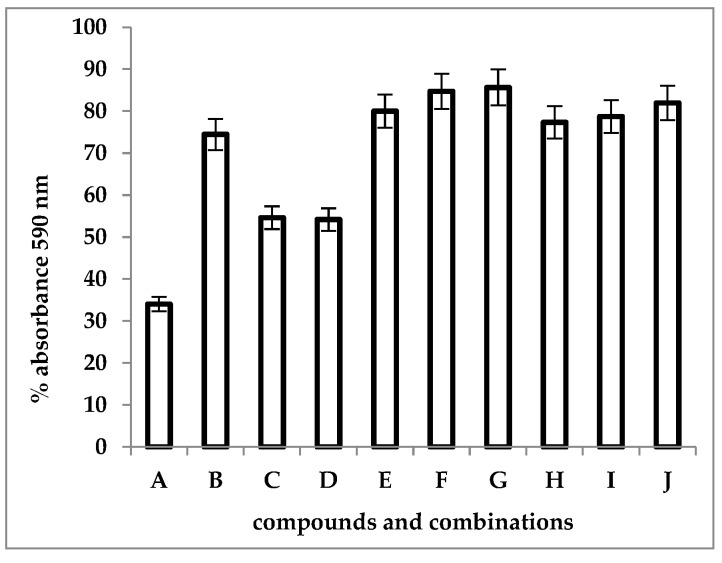
Intracellular uptake of CV in *C. albicans* cells after exposure to compounds and their combinations with SDS. A—control (cells without compounds); B–D—CV uptake by *Candida* spp. cells in the presence of compounds alone (B—SDS 1000 μg/mL; C—SAM3 1 MIC; D—SAM5 1 MIC); E–G—CV uptake by *Candida* spp. cells after exposure to SAM3/SDS combination at increasing concentrations of SAM3 (E—SAM3 1 MIC + SDS; F—SAM3 2 MIC + SDS; G—SAM3 4 MIC + SDS); H–J—CV uptake by *Candida* spp. cells after exposure to SAM5/SDS combination at increasing SAM5 concentrations (H—SAM5 1 MIC + SDS; I—SAM5 2 MIC + SDS; J—SAM5 4 MIC + SDS).

CV uptake was important when SAM3 and SAM5 were used alone, compared to the control. The two compounds showed an important effect of destabilizing the membrane structure, highlighted by the increased intracellular influx of CV. However, their effect on CV uptake when used alone was lower compared to that of SDS, a compound known for its action on the cell membrane. CV influx inside *Candida* cells when the compounds were associated with SDS (Figure 6) was higher as the concentration of imidazoles increased from 1 MIC to 4 MIC, and CV uptake was more intense in the case of SAM3 + SDS than for the SAM5 + SDS combination.

## 4. Discussion

**Figure 7:**
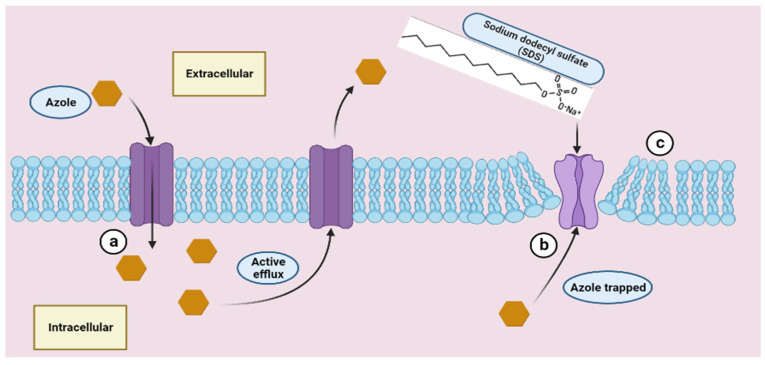
The putative mechanism of imidazoles/SDS synergistic interaction. (**a**) Imidazole molecules enter the cells, but most are removed with functional efflux pumps. (**b**) SDS induces a conformational change in the efflux pumps and inactivates them; therefore, azoles are trapped intracellularly and achieve a lethal concentration. (**c**) SDS can also induce, as a secondary mechanism, a minimal local destabilization of the membrane phospholipid layer, which leads to an additional increase in imidazole influx.

SDS induces changes in carbon metabolism and initiates the signaling pathway of cell wall integrity [34,35]. Its toxicity is mainly due to the disruptive action on the cell wall and membrane permeability [36]. The change in membrane permeability leads to the disturbance of ionic balance and can cause cell lysis [34,35]. These effects were reported at higher concentrations than those used in our experiments. The concentrations used by other authors in different combinations that proved effective varied between 0.5% [22], 1% [37] and 2% [38]. Therefore, we consider that, in our experiments, SDS mainly enhanced the penetration of benzimidazoles inside cells and facilitated their activity, without a significant antifungal activity per se. SDS’s ability to form micelles and emulsions was practically exploited in different combinations. It has been used in many hygiene products (liquid soaps, shampoos and toothpastes), as well as in some food and pharmaceutical products [39]. The association of SDS with different organic or inorganic compounds has been used to control the growth of pathogens in various practical applications. The mixtures of SDS with organic acids, levulinic acid [37,40] and lactic acid [38] have been used for the control of pathogens in food products. The density of *Salmonella enterica* was significantly reduced on the skin of broilers after using a mixture of SDS and organic acids as a decontaminant [41]. Similar results were reported by [22], who noted an increase in the bactericidal efficiency of chlorine and peracetic acid after their supplementation with SDS. The above authors used these mixtures for the control of pathogens in chicken carcasses. Also, there were attempts to add SDS to some natural extracts, where an increase in their antimicrobial efficiency was observed [42]. All these reports showed that SDS improved the antimicrobial effect of some compounds that could thus act more effectively as disinfectants, decontaminants and pathogen control agents. There are several mechanisms that might explain the enhanced activity of imidazole derivatives when associated with SDS. First, SDS might favor a micellar state of imidazoles within hydrophilic environments due to its surfactant properties. SDS can reduce the interfacial tension between two substrata (hydrophobic and hydrophilic) and enhance their self-assembly into micelles [39]. It was observed that eugenol-loaded nanoemulsions manifested improved antifungal activity in conjunction with a possible synergistic effect of SDS [43]. Also, SDS increased the inhibitory activity of some photosensitizers against planktonic *Candida* cells via reducing the tendency of molecules’ aggregation [44]. A similar mechanism could be envisaged for the interaction of SDS with benzimidazoles during our experiments. Secondly, SDS can increase the permeability of cell membranes and thus it enhances the intracellular transport of imidazole derivatives. It is well-known that SDS disrupts cell membranes and activates cell wall integrity signaling, restricting the growth of yeasts [45]. Thirdly, under the form of emulsion, the imidazole derivatives can more easily reach the target structures on which they can exert their inhibitory effect. SDS interferes mainly with mitochondria function and hyphal development in *Candida* spp. [46]. It can be assumed that SDS increased the intracellular concentration of imidazoles and helped their delivery to specific site targets. The ability of SDS to make emulsions with hydrophobic substrates (such as benzimidazoles) can improve their capacity to interact with cells. Thus, this could explain their increased inhibitory efficiency when used in combination with SDS. A defense mechanism of *Candida* spp. Against azoles relies on the altered diffusion of these molecules inside the cells [47]. Azoles inhibit the growth of fungi via decreasing the ergosterol content of the membrane, followed by its destabilization and cell lysis [48]. *Candida* cells have the ability to control membrane fluidity via changing the phospholipids composition ratio [49]. This property gives *Candida* cells a certain degree of resistance to azoles because the decrease in membrane permeability leads to a decrease in the influx of azoles inside the cells. Another more important mechanism relies on the ability of fungi to remove azoles outside the cell by using specific efflux pumps, namely the ABC and MFS proteins [50,51,52,53]. Specific inhibitors can have a direct impact on the activity of membrane-bound pumps [54]. Due to the importance of efflux pumps for fungal cell resistance, the inhibition of active efflux with compounds without antimicrobial function itself could play an important role in reducing azole resistance by using synergistic combinations [54]. Our experiments demonstrated that SDS at very low concentrations that do not affect cell viability can increase the inhibitory effect of some azole compounds. The synergism resulting from the association of imidazoles and SDS could be explained as a consequence of SDS activity at two levels: (a) via the minimal destabilization and localized permeabilization of the cell membrane, unable in itself to affect cell viability, but able to increase the influx of imidazoles; (b) via changing the efflux pumps efficiency which allows the accumulation of a higher amount of imidazoles inside the cells (Figure 7).

The hypothesis shown in Figure 7 is in agreement with several reports that showed that SDS, at low concentrations (which do not affect cell viability), leads to a local destabilization of the membrane structure and an increase in its permeability [54,55,56,57,58]. Therefore, it can be assumed that SDS interferes with the transport mechanism of imidazoles and facilitates their influx inside *Candida,* where they can exert their more pronounced inhibitory effect. The experiments were carried out on planktonic cultures, so extrapolation is very difficult for the effect of these combinations on *Candida* biofilms. Microbial biofilms are highly organized communities made up of active living cells, dead cells, cellular debris, organic matter and inorganic matter, all embedded in a characteristic matrix attached to a substrate [59]. Biofilms, including those formed by *Candida*, show increased resistance to antimicrobials and are more difficult to eradicate when they are involved in infections [59]. Therefore, the results of this study regarding the effect of the association of imidazoles with SDS are limited to free-living *Candida* cells, not attached to the substrate. It would be expected that the association of imidazoles with SDS would have a reduced effect on the viability of cells attached to substrates, as biofilms similar to antimicrobials require higher concentrations to be effective. Additional studies are needed to expand the range of molecules with surfactant properties and to find new combinations with antifungals, especially those more effective in combating infections caused by *Candida* biofilms. Our experiment showed that although potentially valuable, the tested imidazole derivatives cannot exhibit the full antifungal potential due to a less effective interaction with the *Candida* spp. cell wall and membrane. Therefore, the practical approach in using benzimidazoles or similar compounds should take into account that their optimal antifungal activity depends on the association with specific surface-active molecules.

## 5. Conclusions

The antifungal activity of imidazole derivatives has dramatically increased when they were associated with SDS. On average, the inhibitory power of imidazoles increased, after their association with SDS, from 5.78 (AM5) to 9.3 times (SAM3) compared to the initial MIC value. This was an important increase in the fungicidal power of the compounds and relied on their increased absorption due to the higher permeability of *Candida* cells under the influence of SDS. 

## Figures and Tables

**Table 1:** Reference strain and clinical isolates of *Candida albicans* used in this study.

Crt. No.	Strain	Observation	Abbreviation
1	*Candida albicans* ATCC 64124	Reference strain	CaATCC
2	*Candida albicans* CaOI1	Clinical isolate, oral infection, resistant to fluconazole, itraconazole	CaOI1
3	*Candida albicans* CaOI2	Clinical isolate, oral infection, resistant to itraconazole, ketoconazole	CaOI2
4	*Candida albicans* CaII1	Clinical isolate, intestinal infection, resistant to voriconazole, miconazole	CaII1
5	*Candida albicans* CaII2	Clinical isolate, intestinal infection, resistant to clotrimazole, fluconazole	CaII2
6	*Candida albicans* CAII3	Clinical isolate, intestinal infection, resistant to voriconazole, miconazole	CAII3
7	*Candida albicans* CaVI1	Clinical isolate, genital infection, resistant to clotrimazole, fluconazole	CaVI1
8	*Candida albicans* CaVI2	Clinical isolate, genital infection, resistant to clotrimazole, fluconazole, voriconazole, miconazole	CaVI2
9	*Candida albicans* CaVI3	Clinical isolate, genital infection, resistant to fluconazole, voriconazole	CaVI3
10	*Candida albicans* CaVI4	Clinical isolate, genital infection, resistant to clotrimazole, fluconazole	CaVI4

**Table 2:** Inhibitory effect of benzimidazole derivatives on *Candida* strains (mm inhibition zones).

Nr. Crt.	Strain	Fluconazole	Compound		
			SAM3	AM5	SAM5
1	*Candida albicans* ATCC 10231	15	8	5	4
2	*Candida albicans* CaOI1	13	10	6	6
3	*Candida albicans* CaOI2	12	9	4	8
4	*Candida albicans* CaII1	11	10	4	8
5	*Candida albicans* CaII2	12	10	3	7
6	*Candida albicans* CAII3	9	6	5	4
7	*Candida albicans* CaVI1	9	5	3	4
8	*Candida albicans* CaVI2	10	8	5	6
9	*Candida albicans* CaVI3	12	10	6	8
10	*Candida albicans* CaVI4	12	10	4	8
**mean**		11.5 ± 1.84	8.6 ± 2.01	4.5 ± 2.17	6.3 ± 1.76

**Table 3:** MIC value of tested compounds against *Candida* strains (µg/mL).

Strain	Fluconazole	Compound			
		SAM3	AM5	SAM5	SDS
*Candida albicans* ATCC 10231	1.0	125	125	125	1250
*Candida albicans* CaOI1	64	62.5	125	125	625
*Candida albicans* CaOI2	64	250	500	500	1250
*Candida albicans* CaII1	64	125	250	125	1250
*Candida albicans* CaII2	128	125	250	125	1250
*Candida albicans* CAII3	128	500	500	500	625
*Candida albicans* CaVI1	128	500	500	500	625
*Candida albicans* CaVI2	64	125	125	125	1250
*Candida albicans* CaVI3	128	62.5	250	500	625
*Candida albicans* CaVI4	128	125	500	125	625
mean	87.9	200	312.5	275	937.5

**Table 4:** Inhibitory activity of SDS/SAM3 association (µg/mL).

Strain	SDS µg	SAM3	FIC Index
*Candida albicans* ATCC 10231	312.5	15.625	0.375
*Candida albicans* CaOI1	312.5	15.625	0.75
*Candida albicans* CaOI2	625	31.25	0.625
*Candida albicans* CaII1	1250	62.5	1.5
*Candida albicans* CaII2	1250	62.5	1.5
*Candida albicans* CAII3	312.5	15.625	0.531
*Candida albicans* CaVI1	312.5	15.625	0.531
*Candida albicans* CaVI2	312.5	15.625	0.375
*Candida albicans* CaVI3	312.5	15.625	0.531
*Candida albicans* CaVI4	312.5	15.625	0.375
mean	406	26.56	
*p*		0.00071	

**Table 5:** Inhibitory activity of SDS/AM5 association (µg/mL).

Strain	SDS	AM5	FIC Index
*Candida albicans* ATCC 10231	625	31.25	0.75
*Candida albicans* CaOI1	156.25	7.81	0.311
*Candida albicans* CaOI2	625	31,25	0.562
*Candida albicans* CaII1	1250	125	1.5
*Candida albicans* CaII2	1250	125	1.5
*Candida albicans* CAII3	312.5	15,625	0.531
*Candida albicans* CaVI1	312.5	15.625	0.531
*Candida albicans* CaVI2	625	31,25	0.562
*Candida albicans* CaVI3	1250	125	1.5
*Candida albicans* CaVI4	625	31.25	0.75
mean	703.125	53.906	
*p*		0.209357	

**Table 6:** Inhibitory activity of SDS/SAM5 association (µg/mL).

Strain	SDS	SAM5	FIC Index
*Candida albicans* ATCC 10231	312.5	15.625	0.375
*Candida albicans* CaOI1	156.25	7.81	0.312
*Candida albicans* CaOI2	625	31.25	0.562
*Candida albicans* CaII1	1250	62.5	1.25
*Candida albicans* CaII2	1250	62.5	1.5
*Candida albicans* CAII3	312.5	15.625	0.531
*Candida albicans* CaVI1	312.5	15.625	0.531
*Candida albicans* CaVI2	1250	62.5	1.25
*Candida albicans* CaVI3	625	31.25	0.562
*Candida albicans* CaVI4	1250	62.5	1.5
mean	734.375	36.71	
*p*		0.042307	

**Table 7:** Pearson product–moment correlation coefficients.

Control	1
SAM3	−0.89
SDS	−0.74
AM5	−0.85
SAM5	−0.85
SAM3 + SDS	−0.85
SAM5 + SDS	−0.93
AM5 + SDS	−0.93

## Data Availability

All data generated or analyzed during this study are included in this published article.
